# MAGIC polymer gel for dosimetric verification in boron neutron capture therapy

**DOI:** 10.1120/jacmp.v8i2.2409

**Published:** 2007-04-30

**Authors:** Jouni Uusi–Simola, Sami Heikkinen, Petri Kotiluoto, Tom Serén, Tiina Seppälä, Iiro Auterinen, Sauli Savolainen

**Affiliations:** ^1^ HUS, Medical Imaging Centre Helsinki University Central Hospital; ^2^ Department of Physical Sciences University of Helsinki; ^3^ Laboratory of Organic Chemistry University of Helsinki; ^4^ VTT Processes Technical Research Centre of Finland Finland

**Keywords:** gel dosimeter, neutron dosimetry, BNCT, MCNP

## Abstract

Radiation‐sensitive polymer gels are among the most promising three‐dimensional dose verification tools developed to date. We tested the normoxic polymer gel dosimeter known by the acronym MAGIC (methacrylic and ascorbic acid in gelatin initiated by copper) to evaluate its use in boron neutron capture therapy (BNCT) dosimetry. We irradiated a large cylindrical gel phantom (diameter: 10 cm; length: 20 cm) in the epithermal neutron beam of the Finnish BNCT facility at the FiR 1 nuclear reactor. Neutron irradiation was simulated with a Monte Carlo radiation transport code MCNP. To compare dose–response, gel samples from the same production batch were also irradiated with 6 MV photons from a medical linear accelerator. Irradiated gel phantoms then underwent magnetic resonance imaging to determine their R2 relaxation rate maps. The measured and normalized dose distribution in the epithermal neutron beam was compared with the dose distribution calculated by computer simulation. The results support the feasibility of using MAGIC gel in BNCT dosimetry.

PACS numbers: 87.53.Qc, 87.53.Wz, 87.66.Ff

## I. INTRODUCTION

Accurate verification of dose distribution is a requisite for effective radiotherapy. Since their introduction,^(^ ^1^ ^,^ ^2^ ^)^ ferrous sulfate (Fricke) and polymer gels have been shown to be useful tools in measuring dose distributions by magnetic resonance imaging (MRI) in special applications of radiotherapy such as intensity‐modulated radiotherapy or stereotactic radiosurgery.^(3)^ Gel dosimeters have also been applied to proton beams,^(^ ^4^ ^,^ ^5^ ^)^ high‐energy carbon ion beams,^(6)^ and epithermal neutron beams, all of which are used in therapeutic applications.^(^ ^7^ ^–^ ^12^ ^)^ The advantages of gel dosimetry include tissue‐like elemental composition, high spatial resolution, capability for three‐dimensional (3D) dose measurements, and possibility of preparing dosimeters of varying sizes and geometries.

Boron neutron capture therapy (BNCT) is a chemically targeted radiotherapy based on the nuclear reaction of the ^10^B isotope with thermal neutrons. An epithermal neutron beam is often used instead of a thermal one as an external radiation source, because epithermal neutrons penetrate deeper into tissue before they become moderated into the thermal energy range. Current dosimeters include activation foils and wires, paired ionization chambers, and thermoluminescent dosimeters, all of which make mapping a 3D dose distribution laborious.^(^ ^13^ ^–^ ^18^ ^)^ The properties of dosimetry gels make these substances suitable for determining 3D dose distributions in large and complex geometries. This approach is advantageous for BNCT: first, for beam dosimetry (experimentally verifying dose distribution in standard phantoms in three dimensions), and second, for verification of treatment planning in complex geometries such as the head and neck.

The absorbed dose in an epithermal neutron beam consists of several dose components, which can be separated into gamma‐ray and neutron dose. Gamma‐ray dose derives from two sources: the gamma rays generated in the irradiated and surrounding materials mostly through neutron‐capture reaction by hydrogen, and the gamma rays present in the incident neutron beam. Neutron dose derives from fast and thermal neutrons. Fast neutrons deposit their energy mainly through recoil protons created in elastic scattering interactions with hydrogen nuclei. The thermal neutron dose is deposited through protons created in the thermal neutron capture reaction by nitrogen. The various components and their relative share of total absorbed dose are of interest because the polymer gel response to absorbed dose depends on linear energy transfer (LET).^(^ ^4^ ^–^ ^6^ ^,^ ^11^ ^)^ Studies have shown that gel response decreases with high‐LET particles.

Gambarini et al. published several papers on the application of the Fricke dosimeter in the dosimetry of epithermal neutron beams.^(^ ^7^ ^,^ ^9^ ^,^ ^12^ ^,^ ^18^ ^)^ That research group reported using Fricke gel dosimeters of varying composition to separate the dose components of the beam.^(18)^ The drawback of Fricke gel dosimeters is deterioration of dose distribution because of diffusion of ferric ions.^(19)^ To preserve spatial accuracy, the time between irradiation and gel imaging must therefore be kept short (1 – 2 hours).^(20)^ Efforts to reduce the ferric ion diffusion have used chelating agents, phantoms with a honeycomb structure, or variations in the gel composition.^(^ ^20^ ^–^ ^22^ ^)^ Polymer gel dosimeters preserve dose distribution better over time and do not require prompt reading.^(^ ^2^ ^,^ ^23^ ^)^ Polymer gel dosimeters known by the acronym MAGIC (methacrylic and ascorbic acid in gelatin initiated by copper) can be prepared in normal‐room atmosphere more easily and faster than BANG‐type gel.^(^ ^24^ ^,^ ^25^ ^)^

In the present work, we studied the response of MAGIC‐type polymer gel dosimeters in epithermal neutron irradiation. A previous gel dosimetry study in epithermal neutron beam had been done with BANG‐3 polymer gel dosimeters.^(10)^ The linear nature of the BANG‐3 response to total absorbed dose in epithermal neutron irradiation was verified, even though part of the dose is induced by high‐LET particles.

In the current study, epithermal neutron irradiation was applied to a MAGIC‐type polymer gel dosimeter in a large cylindrical quartz glass container. In addition, gel from the same production batch was used to prepare dosimeters in smaller Pyrex glass containers. For dose–response comparison, these smaller containers were then irradiated in the 6 MV photon beam of a medical linear accelerator. The magnitude of the response was determined by using MRI to obtain R2 relaxation rate maps. The measured and normalized dose distribution was compared to the dose distribution calculated by computer simulation.

## II. MATERIALS AND METHODS

### A. Gel preparation

The gel was prepared in normal room atmosphere in a fume cupboard using the gel preparation method described by Fong et al.^(24)^ The chosen MAGIC gel composition was previously used and characterized by Gustavsson et al.^(^ ^25^ ^,^ ^26^ ^)^ Table 1 shows the composition.

To prepare 2.3 kg of the gel, we used gelatin (swine skin, 300 Bloom: Sigma Aldrich Finland, Helsinki, Finland), methacrylic acid (purity grade 99%: Sigma Aldrich), ascorbic acid (minimum 99%: Sigma Aldrich), copper sulfate (pentahydrate, minimum 98%: Sigma Aldrich), and pure deionized water. First, gelatin was mixed with room‐temperature water and stirred for about 30 minutes while heating to 45 ±C. After the gelatin had melted, the mixture was allowed to cool to 35 ±C, when ascorbic acid, copper sulfate, and methacrylic acid were added. The mixture was stirred continuously with a magnetic stirrer.

**Table 1:** Gel components for 1000 g of MAGIC gel

Component	Weight (g)
Gelatin (300 bloom)	82
Methacrylic acid	90
Ascorbic acid	0.352
Copper sulfate	0.02
Water	828

Gel was poured into two Pyrex glass vials (diameter: 4 cm; length: 28 cm; wall thickness: 0.13 cm) and one cylindrical quartz glass container (diameter: 10 cm; length: 21 cm; wall thickness: 0.3 cm). A quartz glass container was used for epithermal neutron irradiation, because Pyrex glass contains known thermal neutron–capturing nuclei.^(10)^

The gel dosimeters were left overnight in dark conditions at room temperature to solidify. The Pyrex glass vials were thereafter closed with screw caps, and the quartz glass container, with a quartz glass lid sealed with Parafilm (American National Can, Chicago, IL). The neutron and photon irradiations took place at 24 and 26 hours post‐manufacture respectively, and MRI at 47 and 45 hours post‐irradiation. This timing accords with the recommendation for MAGIC‐type gels to wait a minimum of 30 hours between irradiation and MRI measurement so that the polymerization process can terminate fully.^(23)^

### B. Photon irradiation

The Pyrex glass vials were irradiated using 6‐MV photons from a Varian 2100 C LINAC medical linear accelerator to absorbed doses of 11.9 Gy and 8.0 Gy at dose maximum. The vials were placed one at a time in a 68×65×56‐cm (widthxlengthxheight) water phantom. The vials were positioned upside down in the phantom with the bottom at the level of the water's surface. The field size was 15×15 cm, and the beam was incident from above. Relative depth dose profile at the beam centerline in the water phantom was measured by scanning with an ionization chamber. The LINAC output per monitor unit was verified in the water phantom by absolute dose measurement using an ionization chamber with calibration traceable to a secondary standard dosimetry laboratory.

### C. Neutron irradiation

**Figure 1:**
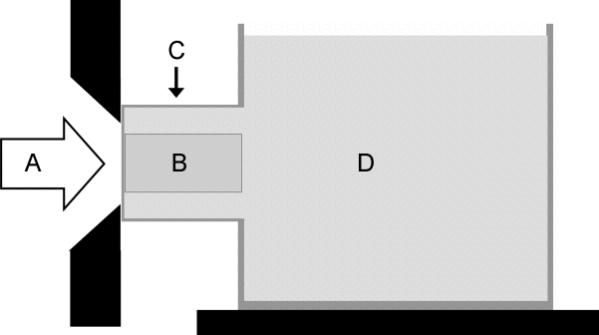
Schematic drawing of the neutron irradiation setup. (A) The epithermal neutron beam is incident from the left through a circular beam aperture. (B) Cylindrical gel container is inserted into (C) a cylindrical extension of (D) the water phantom, which is placed adjacent to the aperture wall.

The quartz glass gel dosimeter was irradiated using the FiR 1 epithermal neutron beam, which is also used for clinical BNCT trials.^(27)^ The dosimeter was placed into a cylindrical extension (diameter and length: 20 cm; wall thickness: 0.5 cm) of a rectangular water phantom that was placed adjacent to the circular 14‐cm–diameter beam aperture as illustrated in Fig. 1. The M55n(n, γ) and A197u(n, γ) activation reaction rates were measured at the beam‐side end of the gel cylinder by diluted Mn–Al (1 wt% Mn) and Au–Al (1 wt% Au) foils (diameter 12 mm; thickness: 0.2 mm), and M55n(n, γ) reaction rates were measured at two sides of the cylinder at 1‐, 2.5‐, 4‐, 6‐, and 10‐cm depths with pieces of Mn–Al wire. The reactor was operated at the power used in clinical irradiation. The beam monitoring system^(28)^ was used to control dose delivery so that the calculated total absorbed dose to the dose maximum was 7.08 Gy (monitor units: N1=140 424 000 counts) at a dose rate of 6.2 Gy/h.

### D. MRI

After irradiation, the gels were stored at room temperature, shielded from light. The MRI analysis was performed using a 1.5 T Siemens Sonata (Siemens Medical Solutions, Erlangen, Germany) 2 days after irradiation. The gels were brought to the MRI room the night before imaging to avoid temperature gradients in the gels. A multi‐echo imaging sequence with a repetition time of 10 000 ms and echo times (TEs) ranging from 25 ms to 800 ms, in 25‐ms steps.

All three gel dosimeters were imaged simultaneously by placing them together in a standard head coil with their centerlines leveled and aligned. In the coronal direction, 11 adjacent slices were acquired such that the middle slice was aligned with the centerlines of the gel dosimeters. The slice thickness was 5 mm; the field of view (FOV), 256×256 mm; and the matrix size, 256×128 pixels. In the axial direction, 12 slices separated by 10‐mm gaps were imaged, the first slice being aligned with the front end of the cylindrical quartz glass container. Slice thickness was 5 mm; FOV, 114×202 mm; and matrix size, 144×256 pixels.

### E. Image analysis

The image analysis was performed with a MATLAB script (Mathworks, Natick, MA) that had been written in‐house. The script calculated the T2 relaxation time maps.

In the analysis, the first echo time (25 ms) was discarded to minimize image artifacts. The first two base images are affected by stimulated echoes and deviate from the mono‐exponential decay curve.^(^ ^29^ ^,^ ^30^ ^)^ The script contained background exclusion by thresholding, followed by a nonlinear fit using the Levenberg–Marquart algorithm^(31)^ to those pixels that did not belong to the background. The effectiveness of the background separation was examined visually. The fitted equation was

(1) $$M_{⊥}(TE)=M_{⊥}(0)\exp\left(\frac{-TE}{T_{2}}\right)\;\;,$$

where $M_{⊥}(TE)$ represents the transverse magnetization at echo time TE, and $M_{⊥}(0)$ corresponds to transverse magnetization at TE 0.

The coronal T2 map at the plane, including the central axes of the two photon‐irradiated gel vials, was converted to an R2 relaxation rate map (R2=1/T2). Values of R2 as the function of depth along the axes of the gel vials were calculated as the mean of 5 adjacent pixels. Subsequently, 22 cm of the gel dosimeter's length was used to determine R2 response as a function of absorbed dose. The absorbed doses ranged from 11.9 Gy to 4.1 Gy and from 8.0 Gy to 2.7 Gy along the length of the vials. A line was fitted to the measured data using a linear least‐squares method.

Coronal and axial R2 maps were used to calculate isodose lines for the neutron‐irradiated gel cylinder. The R2 value corresponding to 0 dose was set according to the linear fit of the photon irradiation results. The maximum R2 value for normalization was determined as the average of a 5×5‐pixel area in the central coronal slice of the cylinder. The same values were used for the axial slices.

### F. Simulation

The Monte Carlo N‐particle transport code MCNP was used to simulate neutron irradiation.^(32)^ The simulation included the 14‐cm diameter FiR 1 beam aperture model, the water phantom, and the gel dosimeter in the quartz glass container. A treatment planning source for neutrons and photons was utilized.^(33)^ Soft‐tissue kerma factors^(34)^ from the International Commission on Radiation Units and Measurements (ICRU) were used to convert neutron fluence into kerma, using dose‐function DE and DF cards from the MCNP for a F4:N neutron fluence tally. Assuming charged‐particle equilibrium inside the phantom, the kerma is approximately equal to the absorbed dose. In a similar manner, the mass energy‐absorption coefficients from the same ICRU report were used to determine absorbed dose attributable to photons, using a F4:P tally for photons. Reaction rates for M55n(n, γ) and A197u(n, γ) were calculated in detector points by using hypothetical materials of pure Au and Mn for tally perturbation and (n, *γ*) reaction (MT=102) in the FM tally multiplier card. Table 2 gives the composition of MAGIC gel and ICRU soft tissue.

**Table 2:** Elemental composition and density of MAGIC gel with 9% methacrylic acid, of International Commission on Radiation Units and Measurements (ICRU) 44 soft tissue, and ICRU 44 brain tissue

Element	MAGIC gel (wt%)	ICRU 44 soft tissue (wt%)	ICRU 44 brain tissue (wt%)
H	10.6	10.2	10.7
C	9.2	14.3	14.5
N	1.4	3.4	2.2
O	78.8	70.8	71.2
Na	—	0.2	0.2
P	—	0.3	0.4
S	0.0003	0.3	0.2
Cl	—	0.2	0.3
K	—	0.3	0.3
Cu	0.0005	—	—
Density (g/cm^3^)	1.06	1.06	1.04

## III. RESULTS AND DISCUSSION

**Figure 2:**
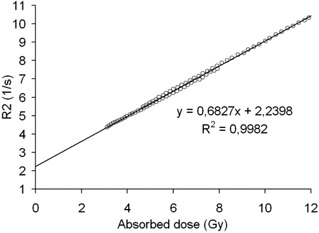
Response of the gels irradiated with 6 MV photons. The least squares method was used to fit a line to the data (equation and correlation coefficient of the fit shown).

Fig. 2 shows the R2 relaxation rates determined for the two photon‐irradiated gel vials as a function of absorbed dose. An average 3% difference in the response between the two gel dosimeters is observed. Given that the dosimeters were made from the same gel batch at the same time and were handled together, the probable cause of the discrepancy may be the result of a slight misalignment (approximately 2 mm) in the irradiation setup for one of the dosimeters.

**Figure 3:**
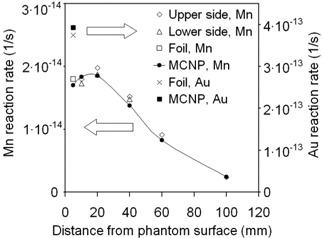
Activation detector measurements and calculations of the epithermal neutron irradiation. Diluted gold (Au, right‐hand scale) and manganese (Mn, left‐hand scale) detectors were both placed on the front side of the gel cylinder, and Mn detectors were placed on the upper and lower sides. The statistical uncertainties related to measurements and Monte Carlo N‐particle (MCNP) calculations are 1.2%−1.6% and 0.1%−1.0% respectively (with geometric uncertainties attributable to irradiation setup or uncertainties in the MCNP source model disregarded).

Fig. 3 collates the results from the activation detector measurements and MCNP calculations for the neutron irradiation. The measurement and calculation points were at the front end of the cylinder and at different depths on the upper and lower surfaces. The calculations and measurements agree within 10%, the largest difference being for Mn wires at 40 mm and 60 mm depth.

**Figure 4:**
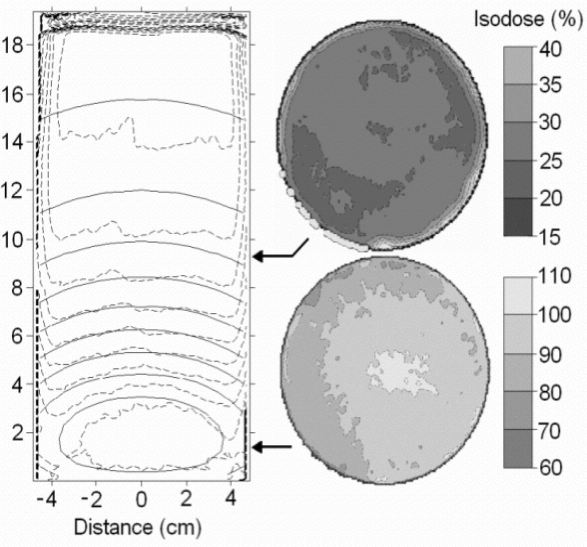
(Left panel) Comparison of calculated (solid line) and measured (dashed line) isodoses (10% intervals, starting from 90% isodose) in the central cross‐section of the cylindrical gel phantom irradiated in the epithermal neutron beam. (Right panel) Two representative axial slice measurements with arrows showing the slice locations in the phantom.

Fig. 4 shows the calculated and measured isodoses for the neutron‐irradiated gel cylinder. The isodose lines calculated by the MCNP correspond to the combined neutron and gamma absorbed dose and were normalized to dose maximum. The longitudinal cross‐section displays better agreement where the radiation dose is highest. The spatial difference of the 30% isodose lines is already more than 1 cm.

**Figure 5:**
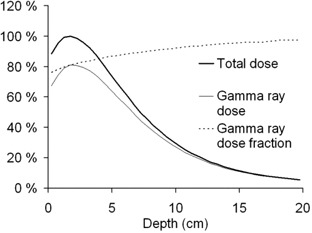
Normalized depth dose curves at the center of the gel cylinder for total and gamma‐ray dose, based on Monte Carlo N‐particle (MCNP) simulation. The fraction of high linear energy transfer neutron dose is highest (20%) at the beam entry and its share decreases with depth.

Fig. 5 shows the calculated normalized depth dose profile at the center of the gel cylinder in the epithermal neutron irradiation. The dose contribution from high‐LET particles is 20% of the total dose near the dose maximum and its proportion decreases with depth.

**Figure 6:**
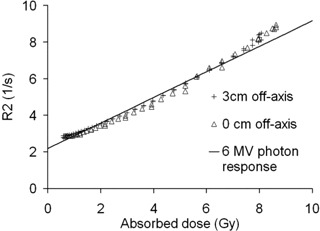
Measured response as a function of the calculated total absorbed dose in the gel cylinder for epithermal neutron irradiation. The measurement points are at the centerline of the cylinder and at 3 cm radial distance from the centerline. For comparison, the solid line shows the fit for photon‐irradiated gels from Fig. 2.

Fig. 6 illustrates the response of the epithermal neutron–irradiated gel as a function of calculated total dose. The points in the figure correspond to the centerline of the gel cylinder and to points at a 3‐cm radial distance from the centerline. The linear fit to the response of the 6 MV photon–irradiated gels is plotted in the figure for comparison. The dose–response appears to be higher at about 8 Gy total dose, where the dose contribution from high‐LET particles is also highest. This finding contradicts our previous results, which showed a decrease in the dose– response of polymer gel to high‐LET radiation. The BANG‐1‐type polymer gel has been reported to underestimate the absorbed dose at the Bragg peak of the proton beam by 20%−30% ^(^ ^4^ ^,^ ^5^ ^,^ ^11^ ^)^ and by 10%−40% at carbon ion beams of various energies.^(6)^ The BANG‐3‐type gels underestimate dose under the same conditions by 50% and 40%−60% respectively.^(^ ^4^ ^–^ 6 ^,^ ^11^ ^)^

These results support the feasibility MAGIC gel in BNCT dosimetry. The properties of the gel make it especially suitable for the determination of 3D dose distribution in large volumes and challenging geometries. The possible uses in BNCT include dosimetric verification of treatment planning in anatomic phantoms and even in vivo dosimetry.

## IV. CONCLUSIONS

The MAGIC‐type polymer gel used in the present study was shown to be feasible for relative‐dose determination in epithermal neutron irradiation. The manufacture and handling of the gel is simple, and a 3D dose distribution can be measured in a large volume with a single irradiation and a single detector. The results show that MAGIC gel dosimetry can be a valuable addition to the available tools in BNCT dosimetry. Further measurements to inspect the high‐LET effect would improve the method.

## ACKNOWLEDGMENTS

The authors thank Mikko Tenhunen for assistance during photon irradiations. The present work was supported by the State Subsidy for University Hospitals, Department of Radiology.
